# Enlargement of the muscle stem cell pool in linc-MYH-deficient mice does not prevent sarcopenia during aging

**DOI:** 10.3389/fcell.2025.1667437

**Published:** 2025-09-24

**Authors:** Qing Yin, Christelle Labib, Thomas Boettger, Thomas Braun

**Affiliations:** ^1^ Department of Cardiac Development and Remodeling, Max-Planck-Institute for Heart and Lung Research, Bad Nauheim, Germany; ^2^ Member of the German Centre for Cardiovascular Research (DZHK), Partner site Rhein-Main, Frankfurt am Main, Germany

**Keywords:** muscle stem cells, sarcopenia, aging, linc-MYH, mice

## Abstract

Loss of skeletal muscle mass and muscle strength during aging (sarcopenia) and reduced skeletal muscle regeneration are often attributed to the age-dependent decline of muscle stem cells (MuSCs). However, it has not been analyzed whether enlargement of the MuSC pool in old animals can attenuate sarcopenia or restore regenerative potential. Here, we directly tested this idea by taking advantage of linc-MYH-mutant mice, which show a substantially increased number of MuSCs in young mice. We found that 24-month-old geriatric linc-MYH knockout mice still maintain a consistently enlarged MuSC pool compared to age-matched controls. MuSCs in geriatric linc-MYH knockout mice were located beneath the basal lamina and remained mostly in a quiescent state. Importantly, enlargement of the MuSC pool did not prevent sarcopenia, or improve muscle function and regeneration. Instead, the larger MuSC pool in geriatric linc-MYH^−/−^ mice resulted in the formation of smaller muscles during regeneration with thicker fibers, characterized by an increased myonuclei content per fiber. Furthermore, we observed shifts of the muscle fiber-type composition in linc-MYH^−/−^ mice during aging, including a reduction of type IIb fibers in the tibialis anterior muscle and a reduction of type IIa fibers in the soleus, combined with an increase of type I fibers.

## Introduction

Sarcopenia is characterized by the progressive loss of muscle mass and strength during aging, compromising physical fitness (Cruz-Jentoft et al., 2019; Sayer and Cruz-Jentoft, 2022). Eventually, sarcopenia causes frailty and diminishes the quality of life. Various factors contribute to sarcopenia, including a sedentary life style, inflammatory conditions and systemic diseases (Tanner, et al., 2015; Sinclair, et al., 2017; Navarro-Cruz, et al., 2019). The dramatic growth of the global aging population has attracted growing attention to the onset, progression and management of sarcopenia.

The maintenance of skeletal muscle mass is supported by muscle stem cells (MuSCs), also known as muscle satellite cells. It is assumed that most MuSCs stay in quiescence under homeostatic conditions until activated by injury or intense physical exercise (Relaix, et al., 2021). Following activation, MuSCs proliferate, differentiate and either form new myofibers or fuse with existing myotubes. The number of MuSCs declines in an age-dependent manner in both mice (Snow, 1977) and humans (Renault, et al., 2002; Kadi, et al., 2004). Comparisons of different fiber types revealed a more pronounced reduction of MuSCs on type II myofibers in humans (Verdijk, et al., 2014). The age-related reduction of MuSCs has been attributed to reduced self-renewal, loss of quiescence, and increased senescence (Renault, et al., 2002; Sousa-Victor, et al., 2014). The correlation between declining MuSC numbers and fiber atrophy suggests that the progression of age-associated sarcopenia might be linked to depletion of the MuSC pool (Brack, et al., 2005; Verdijk, et al., 2010; Sousa-Victor, et al., 2014). However, other studies challenge this notion: diphtheria toxin-mediated depletion of MuSC does not induce sarcopenia (Fry, et al., 2015), and even near complete ablation of MuSCs (>97%) does not reduce the average cross-sectional area of myofibers in aged mice (Keefe, et al., 2015). Gain-of-function approaches, which assess the impact of experimentally increased numbers of MuSCs on sarcopenia, are missing so far.

In our previous work, we identified the long non-coding RNA linc-MYH as a key regulator for limiting the size of the MuSC pool in adult mice, thereby preventing myofiber hypertrophy (Schutt, et al., 2020). Deletion of linc-MYH, which is co-expressed together with the myosin gene cluster, increases the number of MuSCs and muscle mass, resulting in fiber hypertrophy in 10-week-old and 8-month-old mice. Here, we wanted to explore whether the expansion of the MuSC pool is maintained in geriatric mice and whether a potentially higher number of MuSCs has effects on the progression of sarcopenia. We describe that the relative expansion of the MuSC pool in linc-MYH-deficient mice compared to controls, is maintained up to 24 months of age. However, the relative higher number of MuSCs in geriatric linc-MYH mutants does not correlate with increased muscle mass, attenuation of sarcopenia, or improved muscle regeneration. These findings suggest that an enlarged MuSC pool alone is not sufficient to prevent the progression of sarcopenia or improve skeletal muscle regeneration during aging.

## Methods

### Mouse models and cardiotoxin administration

DNA sequences coding for both isoforms of linc-MYH (AK010044 and AK079404) were deleted from the mouse genome by homologous recombination and subsequent removal of the neomycin resistance cassette as previously described (Schutt, et al., 2020). All animal experiments were conducted in compliance with German animal protection laws and were approved by the local animal protection committee of the state of Hessen (ref. B2/2008).

Cardiotoxin (C9759, Sigma) was prepared in 0.9% saline solution at a concentration of 0.06 mg/mL and administered into the tibialis anterior (TA) muscles after induction of anesthesia using 5% isoflurane, followed by maintenance with 2% isoflurane. A total of 50 µL of cardiotoxin was injected evenly into the TA muscle using a 30G needle. Postoperative analgesia was accomplished by providing animals with metamizole-containing drinking water (200 mg/kg/day) beginning 1 day prior to treatment and continuing for 5 days postoperatively. The animals were housed for either 2 or 4 weeks after administration of cardiotoxin to allow muscle regeneration to occur before tissue collection.

### Immunofluorescence staining

Freshly dissected muscles were snap-frozen in liquid nitrogen cooled isopentane for preparation of 10 µm cryosections. Tissue sections were air-dried at room temperature and subsequently fixed in 4% paraformaldehyde for 7 min. Sections were permeabilized in 0.3% Triton X-100/PBS and blocked with 1/10 Blocking One/PBS (Nacalai 03953-95). For experiments involving mouse primary antibodies, the Mouse-on-Mouse Blocking Reagent (Vector Labs, MKB-2213) was applied. Sections were washed three times for 10 min each in 0.01% Triton X-100/PBS. Primary antibody staining was performed by incubating sections overnight at 4 °C with the following primary antibodies: mouse anti-PAX7 (MAB1675, R&D Systems), rabbit anti-Laminin (L9393, Sigma), or rabbit anti-CalcR (AHP635, Bio-Rad) diluted in solution A (02272-74, Nacalai Tesque). The next day, sections were incubated with secondary antibodies (goat anti-mouse IgG1 Alexa Fluor 488, A21121, Invitrogen; goat anti-rabbit IgG Alexa Fluor 594, A11012, Invitrogen) in solution B (02297-64, Nacalai Tesque) for 1 h and washed twice using 0.01% Triton X-100/PBS. DAPI (10236276001, Sigma-Aldrich) was applied at a 1:1,000 dilution in PBS to stain DNA. Finally, sections were embedded using Fluoromount W (21634.01, Serva).

Identification of skeletal muscle fiber types was performed by a combination of antibodies against MyHC type I (BA-D5, DSHB), MyHC type IIA (SC-71, DSHB), and MyHC type IIB (BF-F3, DSHB). Secondary antibodies were goat anti mouse IgG Fcγ 2b DyLight405 (Jackson Immuno, 115-475-207), goat anti mouse IgG Fcγ 1 Alexa 488 (Jackson Immuno, 115-545-205), and goat anti mouse IgM Alexa594 (Jackson Immuno, 115-85-075). The concentration of primary antibodies and secondary antibody were 1:100 and 1:500 respectively. Zeiss Axio Observer and Nikon BioPipeline Slide Scanner were used for image acquisition.

### MuSC isolation and culture

Skeletal muscles were dissected from 24-month-old mice and minced utilizing a Mcllwain Tissue Chopper (TC752). Enzymatic digestion was performed with Dispase (Corning #354-235) and Collagenase II (Worthington #LS004177) for 1 h to dissociate tissue fragments into single cells. The suspension was sequentially filtered through 100 μm, 70 μm, and 40 μm cell strainers, after which red blood cells were removed using 1× red blood cell lysis buffer. Fluorescence-activated cell sorting (FACS) was carried out using anti-CD45-APC (eBioscience #17-0451), anti-CD31-APC (eBioscience #17-0311), anti-Ly-6A/E-APC (eBioscience #17-5981), and anti-integrin-FITC (MBL #K0046-4) antibodies, as previously described (Schutt, et al., 2020). Sorted cells were seeded at a density of 40,000 cells per well in 24-well plates and imaged using the Cellcyte X system.

### Acquisition of MRI data

Magnetic resonance imaging (MRI) was used as a non-invasive method to analyze mouse body composition, enabling *in situ* quantification of fat and muscle mass. Body composition measurements were conducted in mice under isoflurane anesthesia at 15, 18, and 24 months of age. MRI settings were implemented as described by (Schweisgut, et al., 2017). Fat and muscle volumes below the bilateral femoral heads were quantified with ImageJ software, using tissue-specific intensity threshold-based segmentation.

### Physical performance tests

Three independent tests were conducted to determine skeletal muscle function of geriatric mice, including the voluntary running wheel test, the Rota Rod test, and the wire hanging grip test. The running wheel test was done as described by (Wust, et al., 2018). Mice were housed individually in cages equipped with a running wheel (303400-RW-V-M-BU, TSE Systems GmbH) for 3 days. Data from the middle 24-h period were averaged and used for analysis. The Rota Rod apparatus from Harvard Apparatus (LE8240) was used for quantifying coordination abilities of mice. The time duration was automatically recorded when mice disengaged from the rotating rod. The grip test, measuring forelimb endurance by allowing mice to hang from a thick wire, was done as described (Peled-Kamar, et al., 1997). The time was recorded when the mice released their grip and fell. For both the Rota Rod and grip tests, measurements were performed three times per day over three separate days to minimize a potential bias.

### Quantification and statistical analysis

Cross-sectional areas of skeletal muscles were measured by the ImageJ plugin ‘Muscle morphometry’ (Sinadinos, et al., 2015). All statistical analysis was done in GraphPad PRISM 10 and all data are presented as mean ± SD. Student’s t-test or 2-way ANOVA test were used for the analysis as indicated.

## Results

### Enlargement of the MuSC pool in linc-MYH mutants is maintained in geriatric mice

**FIGURE 1:**
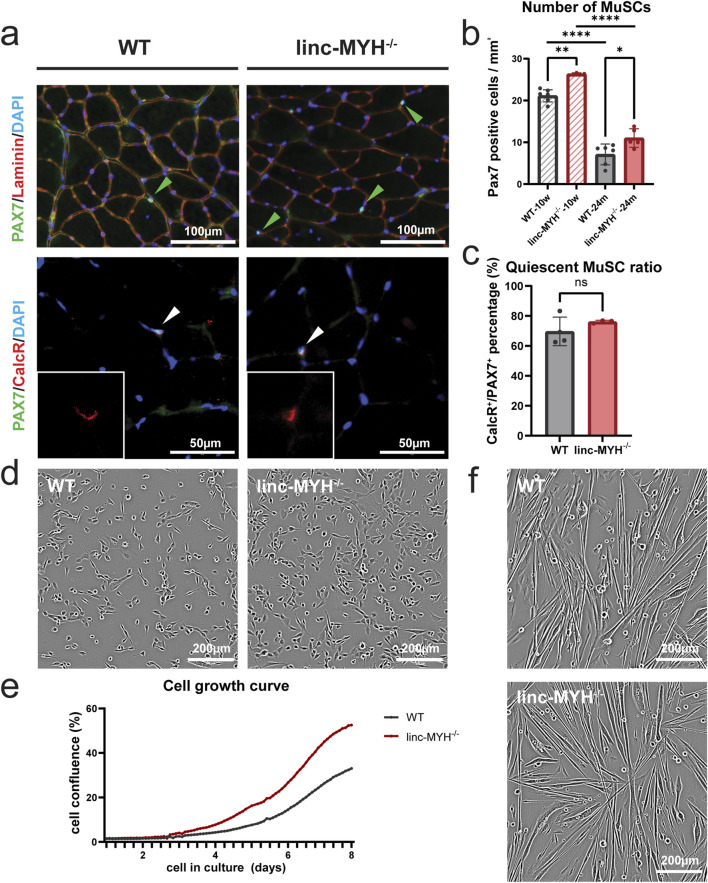
The MuSC pool is enlarged in skeletal muscles of geriatric linc-MYH^−/−^ mice. **(a)** Representative transversal sections of TA muscles of 24-month-old WT and linc-MYH^−/−^ mice, showing PAX7^+^ MuSCs or PAX7^+^/CalcR^+^ quiescent MuSCs. **(b)** Bar graphs depict the mean number of MuSCs per mm^2^ in TA muscles of 10-week-old and 24-month-old mice (10-week: n = 7 WT, n = 3 linc-MYH^−/−^; 24-month: n = 6 WT, n = 5 linc-MYH^−/−^). **(c)** Bar graphs depict the ratio of quiescent MuSCs in 24-month-old mice (n = 4 WT, n = 4 linc-MYH^−/−^). Data are presented as mean ± standard deviation and analyzed by one-way ANOVA and two-tailed Student’s t-test, *p < 0.05, **p < 0.01, ****p < 0.0001. **(d,e)** Representative images of isolated, proliferating MuSCs isolated from 24-month-old mice. Cell proliferation was monitored by time-laps imaging over 8 days (n = 3 WT, n = 2 linc-MYH^−/−^). **(f)** Phase contrast images of differentiating WT and linc-MYH^−/−^ MuSCs 2 days after induction of differentiation. No apparent differences between WT and linc-MYH^−/−^ MuSCs was observed.

Deletion of linc-MYH substantially increases the number of MuSCs in 10-week-old and 8-month-old mice compared to controls (Schutt, et al., 2020). To determine whether aging reduces the differences in MuSC numbers between linc-MYH mutants and controls, we quantified the number of MuSCs in 24-month-old mice by immunostaining for the MuSC marker PAX7. Linc-MYH^−/−^ mice showed a 50% increase in MuSC numbers at 24 months of age relative to age-matched wild-type (WT) controls (WT: 7.09 ± 2.48/mm^2^, linc-MYH^−/−^: 11.01 ± 2.24/mm^2^), consistent with the increase observed at 8 months (Schutt, et al., 2020) (Figure 1a). The number of PAX7-positive MuSCs in 24-month-old linc-MYH^−/−^ mice was significantly lower than in 10-week-old mutants, essentially recapitulating the reduction of the MuSC pool in WT mice, but leaving approximately 50% more MuSCs present (Figure 1b). The similar pattern of depletion suggests that the enlarged MuSC pool of linc-MYH mutants is diminished by comparable mechanisms as in WT controls. We also performed co-staining for PAX7 and Calcitonin Receptor (CalcR), a marker for MuSC quiescence, whose expression decreases during aging together with a reduction of MuSC numbers (Zhang, et al., 2019). Akin to the situation in 10-week-old mice (Schutt, et al., 2020), linc-MYH^−/−^ mice at 24 months of age maintained MuSC quiescence at a comparable ratio to WT (WT: 69.65% ± 9.56%, linc-MYH^−/−^: 76.13% ± 1.00%) (Figure 1c). Furthermore, MuSCs from 24-month-old linc-MYH^−/−^ mice showed an increased proliferation rate compared to WT MuSCs, similar as at 10 weeks, whereas differentiation was normal (Figures 1d–f), consistent with our findings in young adult mice (Schutt, et al., 2020). We concluded that linc-MYH^−/−^ mice represent a suitable model for investigating the impact of an enlarged MuSC pool on sarcopenia and muscle regeneration in aging.

### Enlargement of the MuSC pool does not attenuate loss of muscle mass during aging

**FIGURE 2:**
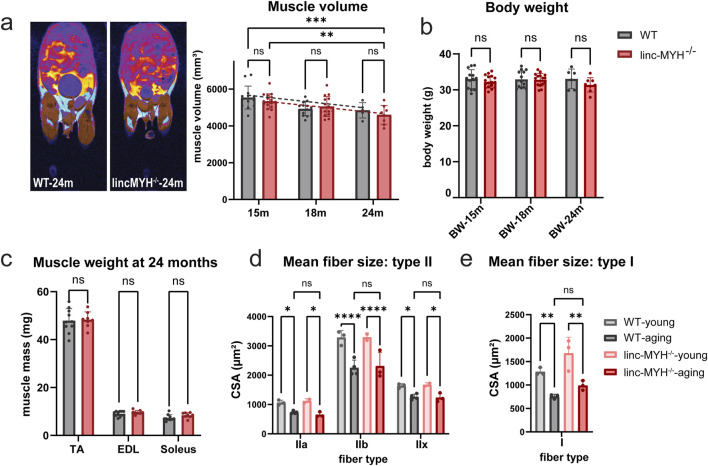
Enlargement of the MuSC pool does not attenuate sarcopenia during aging. **(a)** MRI cross-sectional images illustrating distribution of fat (cyan) and skeletal muscles (brown) in aging mice. Only the lower body was included for the statistical analysis. The bar graphs show mean volumes of muscle tissues at 15, 18, and 24 months (15-month: n = 13 WT, n = 17 linc-MYH^−/−^, 18-month: n = 13 WT, n = 15 linc-MYH^−/−^, 24-month: n = 5 WT, n = 8 linc-MYH^−/−^), **p < 0.01, ***p < 0.001. **(b)** Body weights of WT and linc-MYH^−/−^ mice at 15, 18, and 24 months (15-month: n = 13 WT, n = 17 linc-MYH^−/−^; 18-month: n = 13 WT, n = 15 linc-MYH^−/−^; 24-month: n = 6 WT, n = 8 linc-MYH^−/−^). **(c)** Muscle weights of tibialis anterior (TA), extensor digitorum longus (EDL), and soleus muscles at 24 months (n = 10 WT, n = 8 linc-MYH^−/−^). **(d,e)** Cross-sectional areas of different fiber types in TA or soleus muscles at 10 weeks (young) and 24 months (aging). Data for type II fibers were obtained from TA muscle (10-week: n = 3 WT, n = 2 linc-MYH^−/−^; 24-month: n = 4 WT, n = 3 linc-MYH^−/−^), whereas data for type I fibers were obtained from soleus muscles (10-week: n = 3 WT, n = 3 linc-MYH^−/−^; 24-month: n = 3 WT, n = 3 linc-MYH^−/−^).

To investigate whether an enlargement of the MuSC pool mitigates loss of muscle mass in geriatric mice, we determined the muscle volume. MRI measurements revealed a significant reduction in skeletal muscle volume between 15 and 24 months of age in both WT mice and linc-MYH mutants, with no significant difference between the two groups at any time point (WT-15m: 5535.92 ± 624.68 mm^3^, linc-MYH^−/−^15 m: 5328.59 ± 412.50 mm^3^; WT-18m: 4,925.15 ± 376.75 mm^3^, linc-MYH^−/−^18 m: 5048.47 ± 543.31 mm^3^; WT-24m: 4,842.20 ± 417.39 mm^3^, linc-MYH^−/−^24m: 4,599.87 ± 514.37 mm^3^) (Figure 2a). Moreover, analysis of the cross-sectional area of myofibers revealed atrophy in type I, type IIa, type IIb, und type IIx fibers in both WT and linc-MYH mutants, with no significant difference between the two groups at 24 months of age (WT-young: IIa: 1,062.32 ± 85.98 µm^2^, IIb: 3285.24 ± 241.27 µm^2^, IIx: 1,636.79 ± 74.84 µm^2^, I: 1,282.30 ± 83.53 µm^2^; WT-aging: IIa: 732.43 ± 46.58 µm^2^, IIb: 2,250.81 ± 265.28 µm^2^, IIx: 1,260.03 ± 93.73 µm^2^, I: 755.77 ± 47.00 µm^2^; linc-MYH^−/−^young: IIa: 1,120.69 ± 78.97 µm^2^, IIb: 3294.65 ± 150.03 µm^2^, IIx: 1,672.34 ± 71.05 µm^2^, I: 1,679.04 ± 338.30 µm^2^; linc-MYH^−/−^aging: IIa: 653.18 ± 89.54 µm^2^, IIb: 2,311.13 ± 474.01 µm^2^, IIx: 1,235.25 ± 149.89 µm^2^, I: 989.55 ± 102.54 µm^2^) (Figures 2d,e). Likewise, body weights did not differ significantly between linc-MYH^−/−^ and WT control mice at 15, 18, and 24 months (WT-15m: 33.00 ± 2.63 g, linc-MYH^−/−^15m: 32.21 ± 1.69 g; WT-18m: 32.90 ± 2.39 g, linc-MYH^−/−^18m: 32.83 ± 1.75 g; WT-24m: 33.05 ± 2.73 g, linc-MYH^−/−^24m: 31.37 ± 2.00 g) (Figure 2b). We also measured the weights of different muscles at the end of the observation period at 24 months, which did not uncover differences between the two cohorts (TA: WT: 47.93 ± 5.00 mg, linc-MYH^−/−^: 48.33 ± 3.27 mg; EDL: WT: 9.08 ± 1.12 mg, linc-MYH^−/−^: 9.66 ± 0.84 mg; Soleus: WT: 7.31 ± 1.29 mg, linc-MYH^−/−^: 8.41 ± 1.10 mg) (Figure 2c).

**FIGURE 3:**
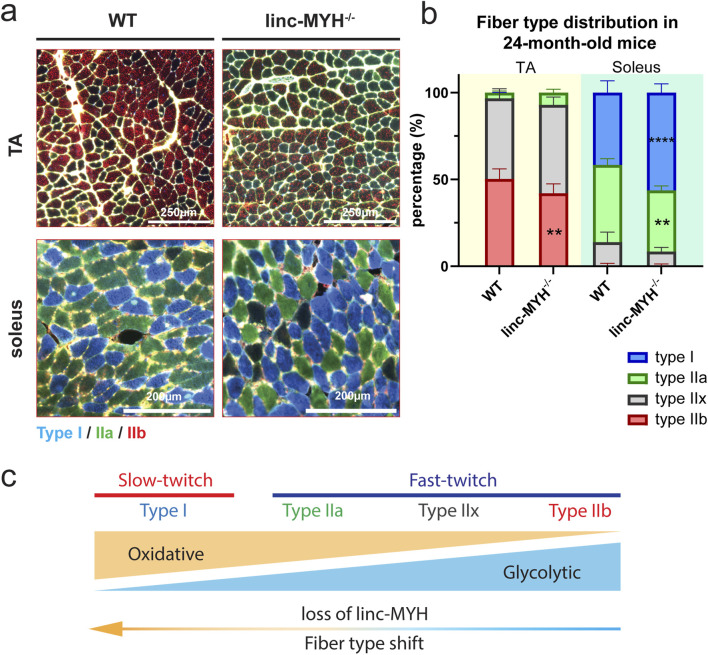
Inactivation of linc-MYH increases the number of slow-twitch muscle fibers in soleus muscles of geriatric mice. **(a)** Representative images of muscle fibers in TA and soleus muscles stained with antibodies against different MyHC isoforms. **(b)** Stacked bar graph showing the proportion of type I, type IIa, type IIb and type IIx fibers in cross-sectioned TA and soleus muscles (n = 4 WT, n = 4 linc-MYH^−/−^), **p < 0.01, ****p < 0.0001. **(c)** Schematic representation of different muscle fiber types and metabolic characteristics. Inactivation of linc-MYH induces a shift toward oxidative muscle fibers.

Atrophy of muscle fiber in aged individuals is most pronounced in type IIA fibers (Murgia, et al., 2017). Since linc-MYH has been described to suppress expression of myosin genes characteristic for slow type I fibers (Sakakibara, et al., 2014), we performed muscle fiber typing using antibodies specific for different myosin heavy chain isoforms. Linc-MYH^−/−^ mice exhibited a shift in fiber type composition at 24 months of age, with a transition from type IIb to type IIa and IIx in the fast-twitch TA muscles, and a shift toward type I fibers in the slow-twitch soleus muscles (Figure 3). Such fiber type switch was not apparent in linc-MYH^−/−^ mice at 10 weeks of age (Schutt, et al., 2020). Since the shift of fiber types in linc-MYH^−/−^ mice was relatively moderate and we observed a decline of more atrophy-prone type IIA fibers, we do not assume that fiber-type differences between WT and linc-MYH^−/−^ mice contribute to the normal progression of sarcopenia.

**FIGURE 4:**
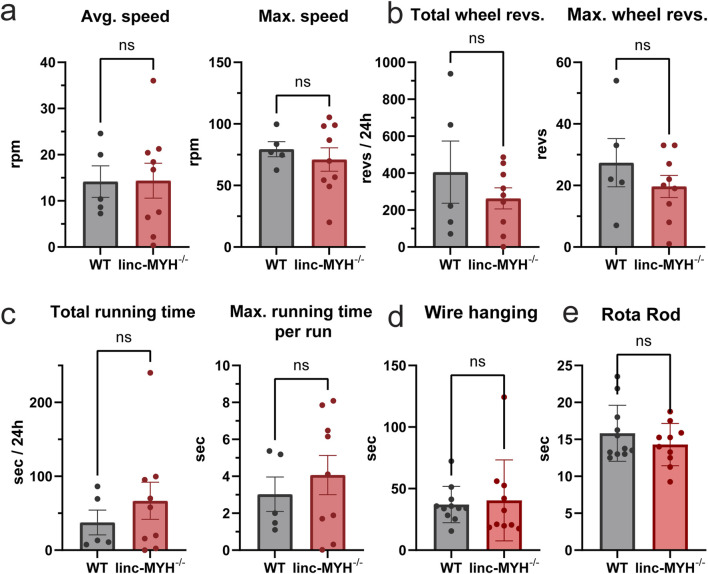
Enlargement of the MuSC pool does not improve muscle functions in geriatric mice. **(a–c)** Quantification of running wheel tests, including average speed, maximum speed, total wheel revolutions, maximum wheel revolutions, total running time and maximum running time per running cycle. Data were continuously recorded over 24 h (n = 5 WT, n = 9 linc-MYH^−/−^). **(d,e)** Quantification of wire hanging and Rota Rod test. Each test was performed three times per day per animal over three consecutive days. The mean value was recorded for each individual (n = 11 WT, n = 10 linc-MYH^−/−^).

Although no significant differences in muscle volume or weight were observed between 24-month-old WT and linc-MYH^−/−^ mice, we next investigated whether the enlarged MuSC pool in linc-MYH^−/−^ mice improves skeletal muscle function in geriatric mice. Several functional tests were employed, including running wheel, wire hanging and Rota Rod tests, which assess physical performance and endurance. No significant differences were observed between WT and linc-MYH^−/−^ mice in the running wheel test (running wheel average speed: WT: 14.17 ± 7.67 revs/min, linc-MYH^−/−^: 14.37 ± 11.35 revs/min; maximum speed: WT: 79.46 ± 13.61 revs/min, linc-MYH^−/−^: 71.02 ± 28.54 revs/min; total wheel revolutions: WT: 405.4 ± 376.7 revs, linc-MYH^−/−^: 263.1 ± 170.9 revs; maximum wheel revolutions: WT: 27.4 ± 17.5 revs, linc-MYH^−/−^: 19.7 ± 10.8 revs; total running time: WT: 37.52 ± 37.38 s, linc-MYH^−/−^: 66.79 ± 75.33 s; maximum running time per run: WT: 3.03 ± 2.08 s, linc-MYH^−/−^: 4.07 ± 3.19 s), wire hanging test (wire hanging endurance time: WT: 37.02 ± 14.75 s, linc-MYH^−/−^: 40.43 ± 32.83 s), and Rota Rod test (Rota Rod endurance time: WT: 15.83 ± 3.79 s, linc-MYH^−/−^: 14.29 ± 2.86 s) (Figures 4a–e).

### The enlarged MuSC pool in 24-month-old linc-MYH^−/−^ mice increases myonuclei number in regenerated myofibers

**FIGURE 5:**
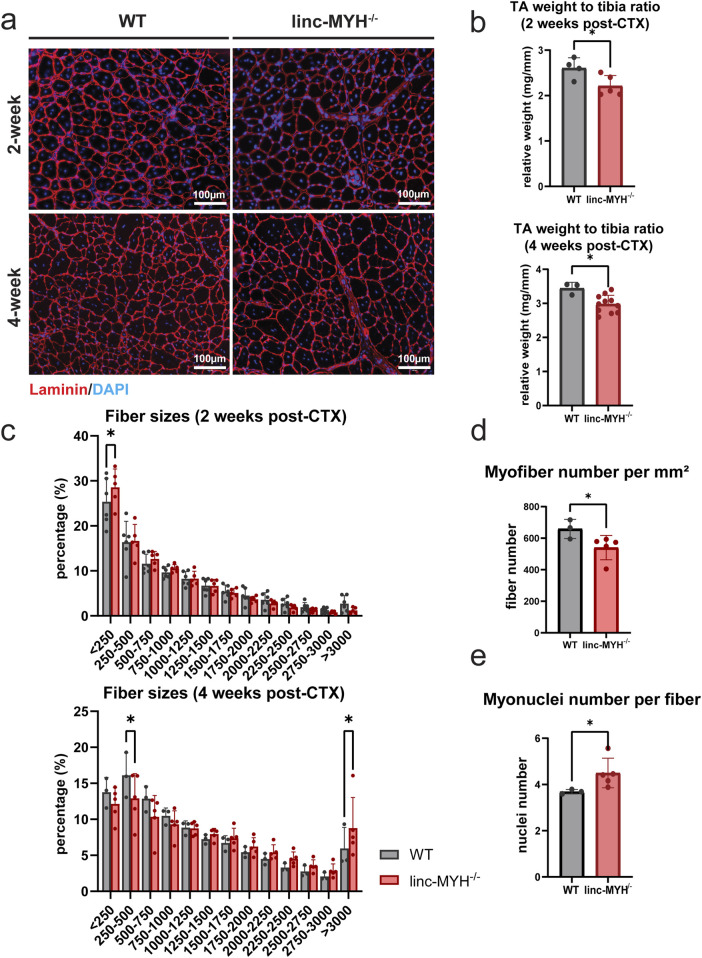
Enlargement of the MuSC pool in geriatric linc-MYH mutant mice is associated with the presence of larger myofibers after completion of muscle regeneration. **(a)** Representative cross-sections of TA muscles after staining for laminin (red) and DAPI (blue), showing newly formed myofibers with centralized nuclei, either 2 weeks or 4 weeks after CTX injection. **(b)** Bar plots comparing relative weights of regenerated TA muscles in WT and linc-MYH^−/−^ mice, either 2 weeks or 4 weeks after CTX injection (2-week: n = 4 WT, n = 5 linc-MYH^−/−^; 4-week: n = 3 WT, n = 10 linc-MYH). **(c)** Distribution of cross-sectional areas (CSAs) of individual TA muscle fibers. Approximately 3,000–5,000 fibers were measured per animal. The x-axis shows CSA values in µm^2^ (2-week: n = 6 WT, n = 5 linc-MYH^−/−^; 4-week: n = 3 WT, n = 5 linc-MYH). **(d)** Quantification of myofiber number per millimeter square, 4 weeks after CTX injection (n = 3 WT, n = 5 linc-MYH^−/−^), *p < 0.05. **(e)** Quantification of myonuclei number per single fiber, 4 weeks after CTX injection (n = 3 WT, n = 5 linc-MYH^−/−^), *p < 0.05.

To test a potential impact of the enlarged MuSC pool in linc-MYH^−/−^ mice for skeletal muscle regeneration in geriatric animals, we performed muscle regeneration experiments by injection of cardiotoxin (CTX). As expected, regeneration was slower in geriatric compared to 10-week-old mice but no obvious differences in the rate of regeneration was detected between WT and linc-MYH^−/−^ mice. However, geriatric linc-MYH^−/−^ mice displayed a higher proportion of thinner myofibers at 2 weeks after CTX injection, followed by a greater number of thicker myofibers at 4 weeks post-injection (Figures 5a,c), consistent with previous findings in 8-month-old linc-MYH^−/−^ mice (Schutt, et al., 2020). Despite the increase of hypertrophic fibers, regenerated muscles of linc-MYH^−/−^ mice showed an overall reduction in muscle weight (WT-2w: 2.61 ± 0.23 mg/mm, linc-MYH^−/−^2w: 2.21 ± 0.23 mg/mm; WT-4w: 3.45 ± 0.17 mg/mm, linc-MYH^−/−^4w: 2.98 ± 0.26 mg/mm) (Figure 5b), as well as a reduction in myofiber density (WT: 658.90 ± 60.93/mm^2^, linc-MYH^−/−^: 540.00 ± 77.14/mm^2^) (Figure 5d). The average number of myonuclei per fiber was increased 4 weeks after injury in linc-MYH^−/−^ skeletal muscles, suggesting that the enlarged MuSC pool in geriatric mice favored the formation of larger fibers rather than contributing to the formation of a larger number of new fibers (WT: 3.69 ± 0.09/fiber, linc-MYH^−/−^: 4.50 ± 0.64/fiber) (Figure 5e).

## Discussion

The decline in MuSC numbers during aging in mice is closely related to the onset of sarcopenia (Brack, et al., 2005; Garcia-Prat, et al., 2016). Furthermore, we recently demonstrated that the depletion of the MuSC pool via inactivation of *Gna12*-*Gna13* or *Rhoa* is associated with accelerated loss of muscle mass during aging (Peng, et al., 2024). In humans the situation is less clear. Although a decline in MuSC numbers during aging of humans was reported (Schmalbruch and Hellhammer, 1976; Renault, et al., 2002; Kadi, et al., 2004; Sajko, et al., 2004), other studies questioned a significant reduction (Hikida, et al., 1998; Roth, et al., 2000). The absence of age-related loss of muscle mass after experimental ablation of MuSCs in mice further complicates the situation (Fry, et al., 2015; Keefe, et al., 2015). In this study, we confirmed a decrease in MuSC numbers and skeletal muscle mass in geriatric mice and demonstrated that an enlargement of the MuSC pool by approximately 50% is not sufficient to attenuate loss of muscle mass. Our data suggest that the size of the MuSC pool alone does not play a pivotal role to slow down the decline of muscle mass during aging. However, our study has some limitations, which need to be considered: (i) The size of the MuSC pool in our model is increased by approximately 50% compared to 24-month-old WT control mice but is still substantially lower than the 10-week-old mice. It is possible that despite the increase of MuSCs in 24-month-old mice the numbers are too low to have an impact on sarcopenia. (ii) The increase of MuSCs in 24-month-old mice was achieved by inactivation of linc-MYH. Absence of linc-MYH favors the pro-proliferative function of the INO80 complex and thereby enlarges the number of MuSCs (Schutt, et al., 2020). We cannot fully exclude that the inactivation of linc-MYH has also negatively effects on MuSCs, limiting their potential anti-sarcopenic functions. However, we did not obtain any evidence that would support such a possibility, since linc-MYH-deficient MuSCs differentiate normally and skeletal muscle regeneration happens in a normal fashion in young linc-MYH mutants. We even observed a modest increase in skeletal mass in young linc-MYH mutants (Schutt, et al., 2020), which was not present any longer in geriatric linc-MYH mutants. Furthermore, the increased size of the MuSC pool in linc-MYH-deficient mice is maintained over the whole lifetime and shows the same percentage of quiescent cells as in controls. Thus, it seems unlikely that the absence of linc-MYH negatively affects MuSCs. In our study we addressed the potential impact of MuSC number on sarcopenia, but it is important to note that alternative mechanisms, such as degeneration of neuromuscular junctions (Gonzalez-Freire, et al., 2014; Iyer, et al., 2021) or metabolic abnormalities (Joseph, et al., 2012; Picca, et al., 2018), contribute to the progression of sarcopenia. The impact of such processes may override putative beneficial effects on sarcopenia, exerted by an increase of MuSCs.

In contrast to missing effects on sarcopenia, the increased MuSC pool had an impact on the size of regenerated muscles in 24-month-old animals. We observed that linc-MYH^−/−^ MuSCs preferentially fused into larger fibers with an increased number of myonuclei. At present, it is hard to judge whether this phenomenon is caused by the enlargement of the MuSC pool or by other intrinsic functions of linc-MYH. The linc-MYH locus is more abundantly expressed in myotubes, particularly in fast-twitch muscles, and in late-stage proliferating MuSCs to terminate proliferation (Sakakibara, et al., 2014; Schutt, et al., 2020; Dos Santos, et al., 2022). In myotubes, linc-MYH participates in myofiber specification by suppressing genes characteristic for slow-twitch fibers (Sakakibara, et al., 2014), which is recapitulated by the increase of type I myofibers in geriatric linc-MYH mutants. It is possible that these additional functions of linc-MYH influence the balance between the formation of fewer and larger myofibers versus the formation of more and smaller myofibers. Despite the limitations of the model system, our data clearly suggest that strategies aimed solely at expanding the MuSC pool are unlikely to succeed in combating sarcopenia. Future research will be needed to determine whether enhancing the function of aged MuSCs, targeting myofiber atrophy, or restoring diminished innervation represent more effective therapeutic approaches.

## Data Availability

The original contributions presented in the study are included in the article/supplementary material, further inquiries can be directed to the corresponding authors.
